# Editorial: Global human identification: studies of its roots, how it may be enlarged, and its expressions in attitudes and behavior

**DOI:** 10.3389/fpsyg.2023.1253525

**Published:** 2023-08-03

**Authors:** Justin Hackett, Katarzyna Hamer

**Affiliations:** ^1^Pennsylvania Western University, California, PA, United States; ^2^Institute of Psychology, Polish Academy of Sciences, Warsaw, Masovia, Poland

**Keywords:** global identity, identification with all humanity, social identity, superordinate identities, globalization, global human identification, global citizenship identification

Research on identity has a long history in psychology and is a thriving area of work within social psychology. Currently, the field is seeing an increase in research and public interest in large, superordinate identities (for a review, see McFarland et al., 2019). Many of the issues confronting humanity today (e.g., climate change, refugee crises, the spread of dangerous diseases such as the SARS-CoV-2, etc.) require a concerted, global effort to solve. As a result, the importance of studying the role of superordinate identification is vital for these efforts.

The idea of such broad identifications is not new. In fact, ideas linking all humanity together were discussed in ancient times by Diogenes of Sinope, Socrates or Chrysippus, and, more recently, by Bartolomé de Las Casas, Baha'u'llah, Thomas Paine, Pablo Casals, Mahatma Gandhi, and many others (McFarland, 2011; Hamer et al., 2019; McFarland et al., 2019). Social identities are also a key feature of many psychological theories. Social Categorization Theory proposes identification with all humanity as the highest possible self-categorization (Turner et al., 1987). In Maslow's (1954) Hierarchy of Needs, self-actualized individuals are described as having developed “a deep feeling of identification, sympathy, and affection for human beings in general”. These individuals feel a deep connection to others and perceive themselves as human first. Adler (1927/1954) described humans as having an innate capacity to care for the wellbeing of other human beings. This can range from concern extending to one's ingroup members to concern for all humanity. According to Allport (1958), without humanity as a common ingroup, human conflict is endless and inevitable. The common theme among these ideas and theories is a bond, or identification, with people around the world.

Work on global social identifications focuses on two approaches: situational, where they are treated as a state, and their level can be experimentally manipulated, or dispositional, where they are treated as rather stable characteristics (see Hamer et al., 2019). Thirteen papers included in this Research Topic of “*Frontiers in psychology*- *global human identification: studies of its roots, how it may be enlarged, and its expressions in attitudes and behavior”*, represent both of these approaches. Building upon earlier research, they present some of the most cutting-edge work on superordinate identifications to date. The collection is very diverse: studies were conducted in Argentina, Canada, Chile, China, England, Germany, India, Iran, Italy, Poland, Russia, Spain, South Africa, and the USA. Together, this work examines the predictors and correlates of global identities (global human identification and global citizenship identification), their potential roots in childhood, as well as their relationship with pressing issues confronting all humanity: attitudes toward migrants, COVID-19, environmental sustainability issues, and international cooperation.

Turmoil around the world, whether due to the impact of war, economic uncertainties, climate crisis, or persecution from one's government, leads to people emigrating in search of a better life for themselves and their families. Migrants are often met with opposition and hostilities. Albarello and Rubini, in an Italian sample, examined psychological factors that lead to denying migrants basic human rights. Perceiving migrants as threatening reduced attributing human rights to them, increased perceptions that one's ingroup is being deprived, and led to lower identification with the human group. Work by Carmona et al. shows that different all-inclusive superordinate categories (e.g., “humans”, “citizens of the world”) differentially impact intergroup helping from host communities toward migrants. Identification with “citizens of the world” was positively associated with opposition to helping migrants and to offering more dependency-oriented helping. In contrast, identification with “humans” was positively associated with greater helping in general and negatively associated with opposition to helping migrants. Grimalda et al.'s experimental work in six countries shows that globally identified individuals cooperate more not only at the global level but more at both the national and local levels as well. With many unprecedented issues confronting humanity requiring novel and global thinking to address, Pastor et al. found in Spanish and diverse migrant samples that identification with a global culture is connected to more creative thinking among host groups. More specifically, they found developing a cultural identity that develops beyond one acquired through enculturation has the potential to facilitate creative behavior.

Several articles in this Research Topic examine the relationship between identification with all humanity (IWAH) and COVID-19. Barragan and Meltzoff found on U.S. samples that IWAH predicted the prosocial motivation to wear masks and, when not wearing a mask, engage in physical distancing in public. Sparkman et al.'s experimental work shows on U.S. samples that manipulating IWAH had a significant effect on participants' psychological bond with all humanity but did not impact their concern for all humanity. Unexpectedly, manipulating IWAH had no causal impact on intentions to engage in behaviors to help attenuate the spread of COVID-19. Wlodarczyk et al. collected data in Spain and Chile at two points during the COVID-19 pandemic and found IWAH significantly decreased in participants over the 2-month course of the strict pandemic lockdowns. This unique project highlights the impact of pandemic exhaustion on people and a corresponding shift to more local concerns and away from global issues. While during the pandemic, there was a general increase in prejudice against people from China, a longitudinal study from Ferrante et al. on a Canadian sample shows that global citizenship identification was connected to the lowest prejudice toward this group.

Two articles in this Research Topic examine the role of superordinate identification and environmental concerns. In a systematic review of 30 articles, Pong and Tam found global identification consistently related to pro-environmental behaviors and environmental concerns. The underlying mechanisms in this relationship—obligation, responsibility, and relevance—underscore the role of human connections and concerns. Urbańska et al., on a Polish sample, explored how perceptions of freedom impact pro-environmental behaviors. Viewing freedom as intrinsic (conditional, limited by the needs of other people) was related to greater environmental concern and pro-environmental behavior than extrinsic one, with IWAH moderating this relationship.

There is still much to learn about the roots of global human identification. Several studies in this Research Topic examine its potential predictors. Hagel et al. on English and German samples found participants' recollections of very general “positive parenting behavior” weakly connected to IWAH, while the extent to which participants perceived their parents as global citizens accounted for a third of the variance in their own identification as global citizens. Hamer and McFarland on U.S. and Polish samples examined childhood and adolescent experiences of being raised in diverse environments, having intergroup friendships, helping or being helped by various others, and having experiences leading to re- or de-categorization processes predicted IWAH beyond other known factors, such as openness to experience, empathy, universalism and others (Hamer et al., 2019), while parental styles did not predict IWAH. Wu et al. examined, on a Chinese sample, the connection between pop culture and IWAH. Specifically, engaging with science fiction—literature, movies, and comics that portray alternative worlds and events—was positively related to IWAH. These programs of research illustrate the ways in which early experiences and pop culture have the ability to make people more open to others and impact global social identifications.

Cumulatively, these studies explore the predictors, correlates, roots, and consequences of global identities in a variety of contexts and countries. As Editors, we would like to express our gratitude to all the contributing authors, and reviewers for their support of this Research Topic.

We began this Research Topic working with another co-editor, our colleague and friend Dr. Sam McFarland who unfortunately passed away in 2022. Dr. McFarland was an incredible teacher, researcher, and mentor. His efforts, openness to everyone, and his groundbreaking work on identification with all humanity, brought researchers from around the world together and began many of the collaborations that evolved into the international Identification With All Humanity Lab. We hope this Research Topic, which we dedicate to him, positively contributes to the ever-evolving legacy of his work.

## Author contributions

JH: Writing—original draft. KH: Writing—review and editing.
